# Unexpected Fatal Pneumocystis Jirovecii Pneumonia During Triplet Therapy for Hormone‐Sensitive Prostate Cancer

**DOI:** 10.1002/rcr2.70435

**Published:** 2025-12-08

**Authors:** Fumihiro Ito, Koki Kobayashi, Gaku Hayashi, Shunsuke Kamijo, Takashi Fujita

**Affiliations:** ^1^ Department of Urology Gifu Prefectural Tajimi Hospital Tajimi Japan

**Keywords:** darolutamide, docetaxel, hormone‐sensitive prostate cancer, opportunistic infection, pneumocystis jirovecii pneumonia

## Abstract

Pneumocystis jirovecii pneumonia (PJP) is a life‐threatening opportunistic infection typically seen in immunocompromised patients. It is rarely reported during standard systemic therapy for prostate cancer. A 75‐year‐old man with low‐volume metastatic hormone‐sensitive prostate cancer (mHSPC) received triplet therapy with relugolix, darolutamide and docetaxel. He had well‐controlled diabetes and no history of immunosuppression. After the second docetaxel cycle, he developed anorexia. Chest CT revealed bilateral ground‐glass opacities. β‐D‐glucan was elevated, and sputum PCR detected P. jirovecii DNA. Despite appropriate trimethoprim‐sulfamethoxazole and corticosteroid therapy, he died of respiratory failure ~10 days after symptom onset. This case highlights an underrecognized risk of PJP during triplet therapy for mHSPC. Prophylaxis should be considered in select high‐risk patients, including elderly individuals or those with metabolic comorbidities.

## Graphical abstract

A fatal case of Pneumocystis jirovecii pneumonia occurred during triplet therapy for metastatic hormone‐sensitive prostate cancer in an elderly patient without classical immunosuppressive factors. Subtle early symptoms led to rapid respiratory decline. Clinicians should consider opportunistic infections and monitor lymphocyte counts in vulnerable patients receiving combination therapy.

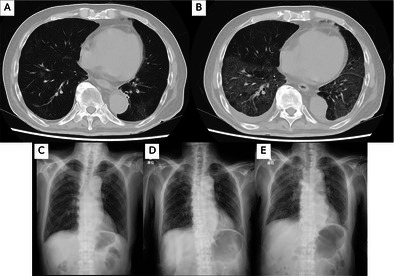

## 1 Introduction

Triplet therapy—combining androgen deprivation therapy, docetaxel and an androgen receptor‐axis targeted agent—prolongs survival in metastatic hormone‐sensitive prostate cancer (mHSPC) [1, 2]. Although Pneumocystis jirovecii pneumonia (PJP) primarily affects patients with HIV or hematologic malignancies, cases in solid tumour patients have been increasingly recognised [3, 4]. We report a fatal PJP case during triplet therapy in a patient without classical immunosuppressive conditions. With triplet therapy rapidly becoming standard practice, recognising rare but severe infectious complications is increasingly important for clinical oncologists.

## 2 Case Report

A 75‐year‐old man was diagnosed with low‐volume metastatic hormone‐sensitive prostate cancer (mHSPC). His PSA level was 342 ng/mL, and imaging revealed a solitary sacral bone metastasis, consistent with low‐volume disease. Bone scintigraphy showed a single sacral lesion with a Bone Scan Index (BSI) of 0.14%, and axial fused SPECT/CT confirmed focal tracer uptake in the sacral bone. He was staged cT4N1M1 and had a Gleason score of 4 + 4 = 8 in 8 of 12 biopsy cores. Pelvic nodal involvement consistent with cN1 was identified on contrast‐enhanced CT, showing bilateral internal iliac lymph nodes measuring ~10 mm in short‐axis diameter.

His medical history included well‐controlled diabetes mellitus (HbA1c 6.7%), hypertension and prior myocardial infarction. He had no history of immunosuppressive conditions, HIV infection or chronic pulmonary disease.

Initial management included percutaneous nephrostomy for post‐renal obstruction. He began oral relugolix (ADT), followed by darolutamide (1200 mg/day) and docetaxel (70 mg/m^2^), with oral prednisolone (10 mg) as premedication.

**FIGURE 1:**
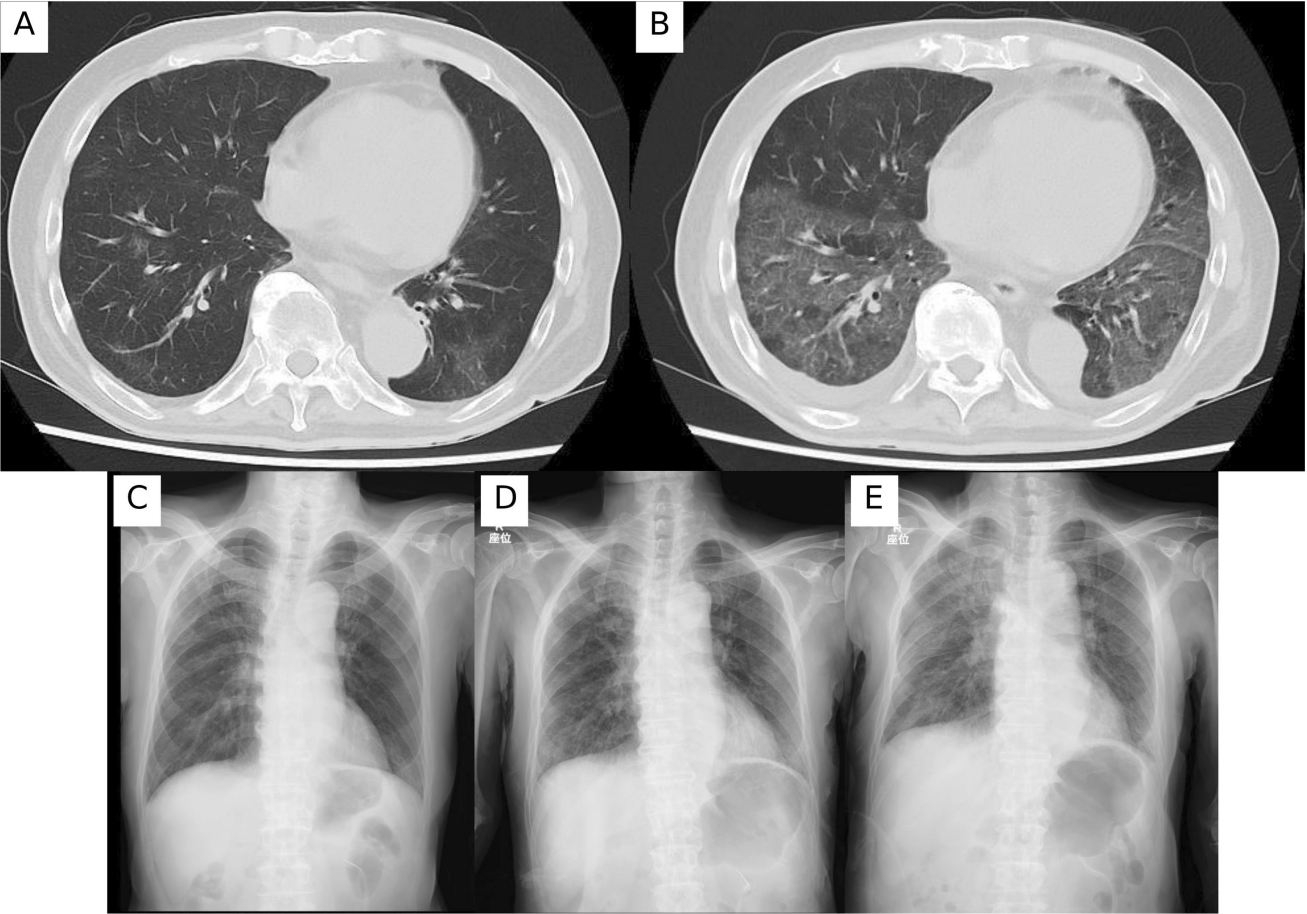
Serial thoracic imaging demonstrating progressive Pneumocystis jirovecii pneumonia. (A, B) Axial chest CT scans on hospital days 1 and 7 showing progressive bilateral ground‐glass opacities and interlobular septal thickening (arrows). (C–E) Upright anteroposterior chest radiographs on hospital days 1, 5 and 9 demonstrating worsening diffuse reticular and ground‐glass opacities consistent with non‐HIV Pneumocystis jirovecii pneumonia.

After the second cycle of docetaxel, he developed anorexia without fever or dyspnea. Chest CT on hospital day 1 showed diffuse, bilateral, non‐segmental ground‐glass opacities and serial x‐rays demonstrated rapid progression of bilateral infiltrates (Figure 1). Serum β‐D‐glucan was elevated, and P. jirovecii DNA was detected in sputum PCR, confirming PJP.

**FIGURE 2:**
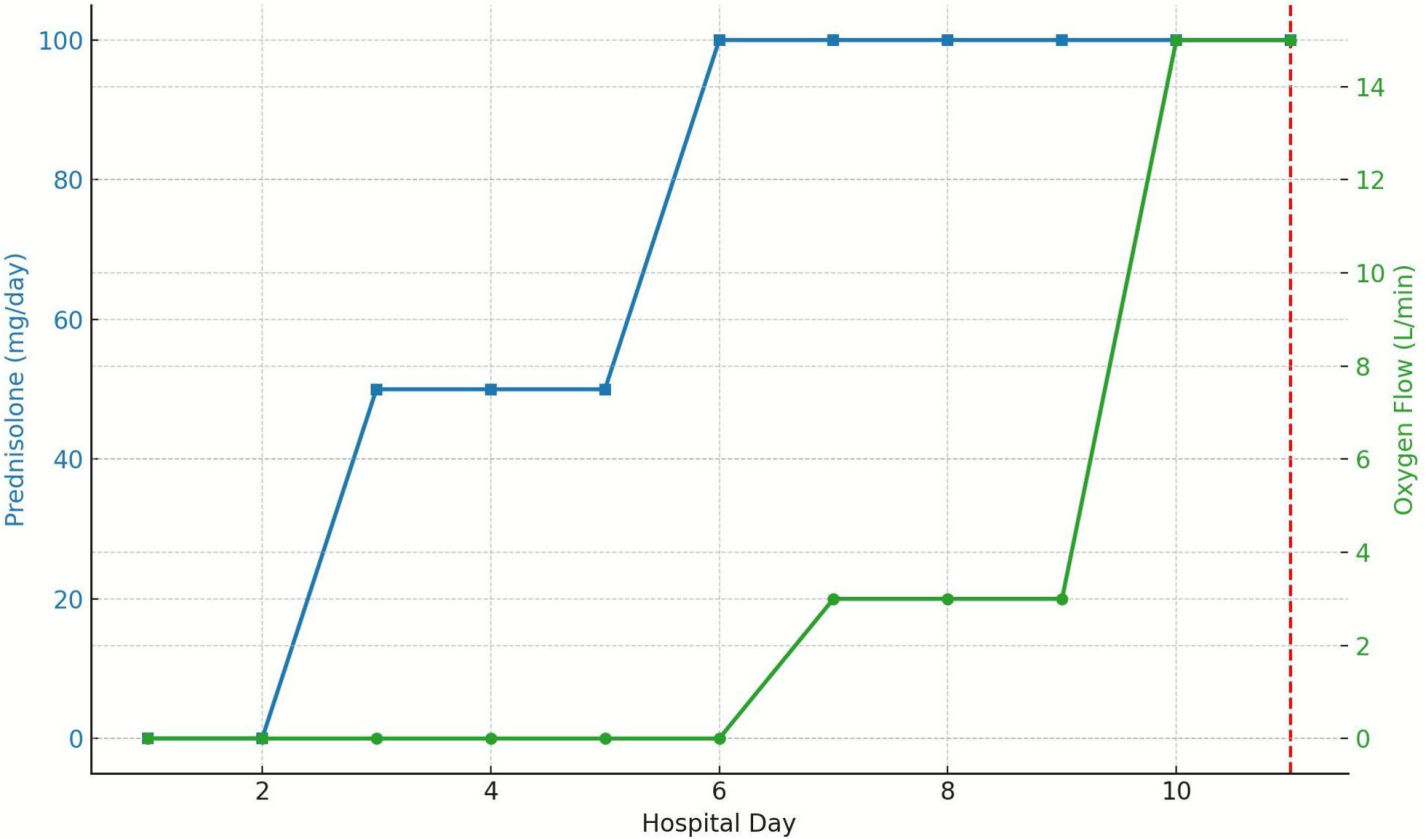
Clinical course of steroid therapy and respiratory status. Changes in prednisolone dose (left *y*‐axis, blue line) and oxygen flow rate (right *y*‐axis, green line) during hospitalisation. Intravenous methylprednisolone was initiated on day 1 and transitioned to oral prednisolone on day 3, with subsequent escalation in response to deteriorating respiratory function. Oxygen supplementation was required from day 7 and rapidly increased thereafter. The red dashed line indicates the day of death.

Despite trimethoprim‐sulfamethoxazole and methylprednisolone treatment, his respiratory function rapidly declined. Intravenous methylprednisolone was initiated on day 1 and transitioned to oral prednisolone on day 3, with subsequent escalation due to deterioration. High‐flow oxygen therapy was introduced on day 7, and he died of respiratory failure ~10 days after symptom onset (Figure 2).

## 3 Discussion

PJP is a rare but potentially life‐threatening opportunistic infection in solid tumour patients, particularly in the absence of classical risk factors such as HIV infection or prolonged corticosteroid exposure [3]. In prostate cancer, PJP is seldom reported and its occurrence during standard systemic therapy such as triplet therapy remains extremely limited [1, 2]. This case highlights that even well‐selected patients may develop severe opportunistic infections during intensified systemic treatment.

Docetaxel is known to induce transient but profound lymphopenia, particularly affecting CD4^+^ T‐cells, which play a central role in host defence against pneumocystis [4]. In elderly patients with metabolic comorbidities such as diabetes, host immune reserve may be further impaired, creating a permissive environment for opportunistic pathogens. Although corticosteroid exposure was short and limited to chemotherapy premedication, it may have contributed synergistically to immune dysfunction. Androgen deprivation may also modulate immune function, although the clinical relevance remains debated. In combination, these factors likely precipitated PJP in this patient despite the absence of overt immunosuppression.

These findings are consistent with prior evidence demonstrating docetaxel‐induced CD4^+^ T‐cell suppression and increased susceptibility to opportunistic infections [5].

Non‐HIV PJP frequently presents with atypical or subtle symptoms, often lacking fever or dyspnea in early stages [5]. Consistent with this pattern, our patient initially presented with nonspecific anorexia, leading to rapid deterioration despite prompt treatment. This underscores the importance of early imaging and fungal testing in patients receiving triplet therapy who develop unexplained constitutional symptoms.

Given the widespread adoption of triplet therapy in mHSPC, prophylaxis against PJP may be considered in selected high‐risk patients, particularly older individuals with comorbidities or chemotherapy‐associated lymphopenia. Practical clinical thresholds have not yet been established; however, monitoring lymphocyte counts and considering prophylaxis when values fall below ~500/μL may be reasonable in vulnerable patients. β‐D‐glucan testing and early CT evaluation in symptomatic patients may further aid early detection.

In conclusion, this case emphasises the need for heightened awareness of opportunistic infections in patients receiving mHSPC triplet therapy and suggests that proactive infection surveillance and prophylaxis strategies may be warranted in select individuals. Non‐HIV PJP carries a higher mortality than HIV‐associated disease, particularly in patients with delayed diagnosis or treatment. With increasing adoption of triplet therapy for mHSPC, prospective data and practical guidance regarding infection surveillance and prophylaxis thresholds, such as lymphocyte counts, are urgently needed.

## Author Contributions

Conceptualisation: Fumihiro Ito. Investigation: Fumihiro Ito, Koki Kobayashi, Gaku Hayashi, Shunsuke Kamijo. Writing – original draft: Fumihiro Ito. Writing – review and editing: Takashi Fujita. Supervision: Takashi Fujita. All authors contributed to the clinical management of the patient and approved the final manuscript. All authors are from the same institution and collaborated directly on patient care and manuscript preparation.

## Funding

The authors have nothing to report.

## Ethics Statement

An ethics statement was not required for this single‐patient case report in accordance with institutional policy.

## Consent

Written informed consent was obtained from a family member who signed the official Respirology Case Reports patient consent form on behalf of the patient, including permission to publish clinical details and all accompanying images.

## Conflicts of Interest

The authors declare no conflicts of interest.

## Data Availability

Data sharing not applicable to this article as no datasets were generated or analysed during the current study.
