# Correction to “Unexpected Fatal Pneumocystis Jirovecii Pneumonia During Triplet Therapy for Hormone‐Sensitive Prostate Cancer”

**DOI:** 10.1002/rcr2.70484

**Published:** 2026-02-02

F. Ito, K. Kobayashi, G. Hayashi, S. Kamijo, T. Fujita, “Unexpected Fatal Pneumocystis Jirovecii Pneumonia During Triplet Therapy for Hormone‐Sensitive Prostate Cancer,” *Respirology Case Reports* 13, no. 12 (2025): e70435, https://doi.org/10.1002/rcr2.70435.

In this published article, the androgen deprivation therapy was incorrectly described as relugolix in the Abstract and the Case Presentation sections.

In the Abstract section, the text “received triplet therapy with relugolix, darolutamide and docetaxel” was incorrect. It should read “received triplet therapy with degarelix, darolutamide and docetaxel”.

In the Case Presentation section, the sentence “He began oral relugolix (ADT), followed by darolutamide and docetaxel” was incorrect. It should read “He began degarelix (ADT), followed by darolutamide and docetaxel”.

We apologise for these errors.

